# Mid-term results of endoscopic mitral valve repair and insights in surgical techniques for isolated posterior prolapse

**DOI:** 10.1186/s13019-023-02352-9

**Published:** 2023-08-18

**Authors:** Enrico Squiccimarro, Vito Margari, Georgios Kounakis, Giuseppe Visicchio, Clemente Pascarella, Crescenzia Rotunno, Carmine Carbone, Domenico Paparella

**Affiliations:** 1 Division of Cardiac Surgery, Department of Medical and Surgical Sciences, University of Foggia, Viale Pinto Luigi, 251, Foggia, 71122 Foggia Italy; 2 Cardio-Thoracic Surgery Department, Heart & Vascular Centre, Maastricht University Medical Centre, Maastricht, The Netherlands; 3 Division of Cardiac Surgery, Santa Maria Hospital, GVM Care & Research, Bari, Italy; 4 Department of Surgical, Medical and Molecular Pathology and Critical Care Medicine, University of Pisa, Pisa, Italy

**Keywords:** Endoscopic mitral valve repair, Minimally invasive cardiac surgery, Long-term results

## Abstract

**Background:**

The adoption of minimally invasive techniques to perform mitral valve repair surgery is increasing. This is enhanced by the compelling evidence of satisfactory short-term results and lower major morbidity. We analyzed mid-term follow-up results of our experience, and further compared two techniques: isolated leaflet resection and neochord implantation for posterior leaflet prolapse.

**Methods:**

Data for all consecutive endoscopic mitral valve repairs via video-assisted right anterior mini-thoracotomy were analyzed between December 2012 and September 2021. The early and mid-term follow-up results were ascertained. The main outcome was the incidence of mortality and the recurrence of significant mitral regurgitation during follow-up which were summarized by the Kaplan-Meier estimator and compared between treatment arms using the stratified log-rank test. Secondary outcomes were the early-postoperative results including 30-days mortality and the occurrence of major complications.

**Results:**

A total of 309 patients were included. Along with ring annuloplasty, 136 (44.4%) patients received posterior leaflet resection (122 isolated) whereas 97 (31.1%) underwent posterior leaflet chords implantation (88 isolated). Forty-nine patients had annuloplasty alone. In-hospital mortality was 1.0%. Mean follow-up was 28.8 ± 22.0 months (maximum 8.3 years). Kaplan–Meier survival rate at 5 years was 97.3 ± 1.0%, mitral regurgitation ($\ge$3+) or valve reoperation free-survival at 5 years was estimated as 94.5 ± 2.3%. Subgroup time-to-event analysis for the indexed outcomes showed no statistical significance between the techniques.

**Conclusions:**

Endoscopic mitral valve repair is safe and associated with excellent short- and mid-term outcomes. No differences were found between leaflet resection and gore-tex chords implantation for posterior leaflet prolapse.

## Introduction

The STS ACSD demonstrates a surge of MVS that doubled in the last decade [1]. In this landscape, the trend of MIMVS in North America has plateaued over the last 11 years, with a share of 20.1% and 24.6% respectively reported in 2010 and 2021 [2]. Data from a recent large multicenter study rather depicts a drastically upward trend of MIMVS, which became the preferred technique currently performed in approximately 70% of cases in dedicated centers [3]. However, despite robust evidence of superior surgical results even as challenging pathology is encountered (i.e., higher repair rates), and lower major short-term morbidity [2], MIMVr is still unestablished as the benchmark against traditional sternotomy nor acknowledged as the technique of choice in guidelines. Among the detractors’ pieces of resistance: safety issues, increased learning curve, prolonged intervention time, feasibility of addressing complex anatomy, and repair durability are advocated [4, 5]. Hereby we analyzed the early and mid-term follow-up results of our endoscopic MVr experience, and further compared isolated leaflet resection versus non-resection techniques to correct PML prolapse.

## Methods

This study was conducted according to the ethical standards of the Declaration of Helsinki, with the need for individual patient consent waived by the Institutional Ethics Committee due to the retrospective nature of our investigation. Notwithstanding, all patients undergoing any cardiac operation at our center are asked to sign a specific informed consent about perioperative and follow-up data collection for research and quality control purposes. Data from our clinical and administrative – prospectively used – database were analyzed from December 2012 to September 2021. Operations were performed by the same group of surgeons. Over 700 operations were performed through a right mini-thoracotomy, including MV replacements, aortic valve replacements, interatrial septum defects closure, and cardiac tumors excisions. In this study we included all consecutive patients who received endoscopic MVr via video-assisted right anterior mini-thoracotomy, including those receiving concomitant TV repair, surgical ablation for AF, closure of either PFO or LAA. Concomitant tricuspid surgery was performed only in cases of regurgitation > 3+, whereas moderate insufficiency was treated depending on annular dilatation. The decision to perform concomitant surgical ablation was rather based on the AF type (i.e., excluding permanent AF) and the left atrial size (i.e., < 50 mm). Emergency operation or any other procedure, including MV replacement was applied as exclusion criteria.

The primary outcome of the study was the incidence of mortality and the recurrence of significant MR during follow-up. Secondary outcomes were the early-postoperative results including 30-days mortality and the occurrence of major complications (e.g., cerebrovascular accidents, thromboembolism, kidney function worsening, permanent pacemaker insertion, reopening for bleeding, POAF, mechanical circulatory support). All major outcomes have been reported according to VARC-2 definitions [6]. Follow-up information was performed at internal outpatient clinics or within territorial health care facilities and obtained by phone contact with the patient.

### Statistical analysis

Data are reported as mean ± standard deviation, median (IQR) or percentage for categorical variables. We used Student’s t-test for intergroup comparison of quantitative variables, whereas either Pearson’s Chi squared test of independence or Fisher’s Exact test were used as appropriate for intergroup comparison of categorical variables. Stratified analyses were performed according to the surgical technique (i.e., isolated PML resection or neochordae implantation). Cumulative survival was evaluated using the Kaplan–Meier method with construction of survival curves, and compared with opposed curves using the log-rank statistic. All reported P-values are two-sided and if < 0.05 were considered statistically significant. The analyses were done with RStudio for macOS (RStudio, Boston, MA, USA).

### Preoperative work-up

The institutional preoperative work-up encompasses transthoracic echocardiography performed by in-house cardiologists, with standardized protocol for the execution of the examination and the interpretation of the results, that are further appraised by Heart Team. Moreover, coronary angiography is performed based on patients’ characteristics, underlying disease, and cardiovascular disease risk profile. In addition, we resort to preoperative CT-scan in cases in which the use of endovascular balloon occlusion of the Aorta (IntraClude, Edwards Lifesciences, USA) is anticipated.

### Surgical technique

**Fig. 1:**
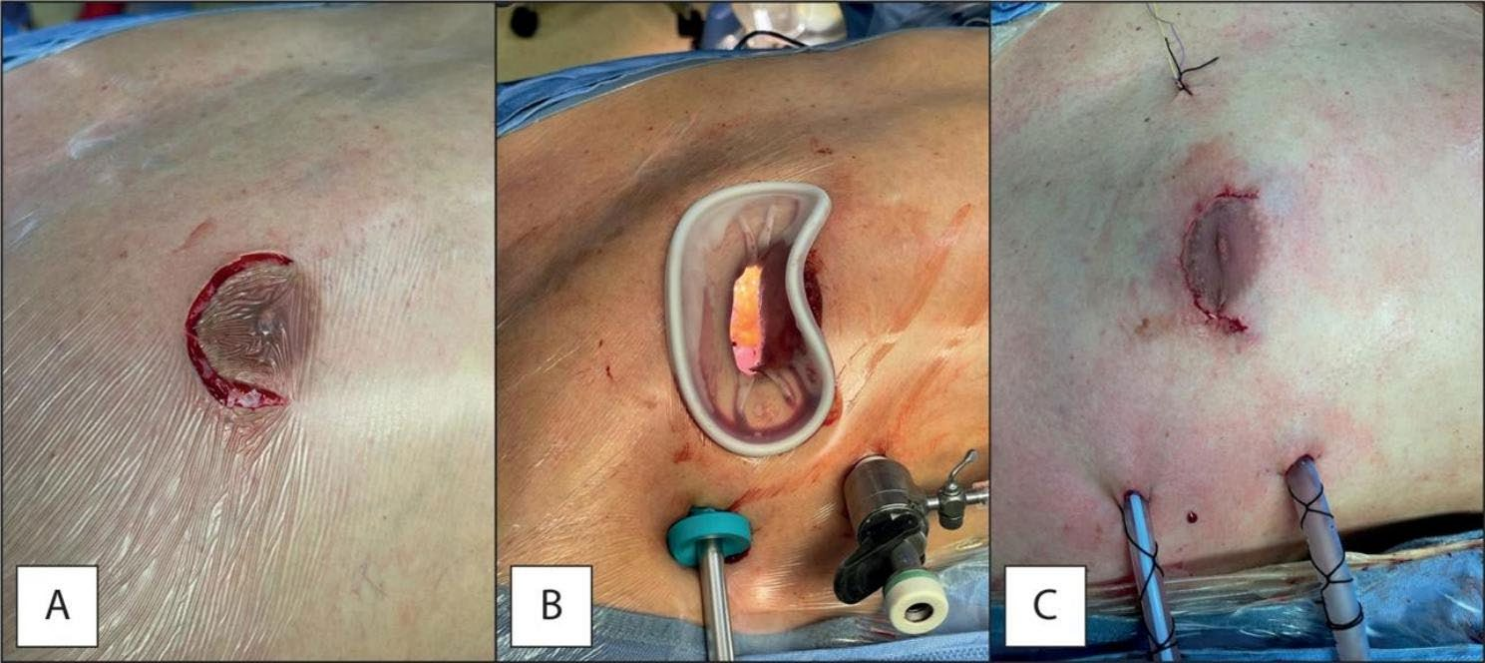
Periareolar approach: incision (Panel **A**), set-up (Panel **B**), final result (Panel **C**)

All patients received endoscopic MVr via a 5 cm right anterolateral mini-thoracotomy at the level of the 3rd or 4th intercostal space, a soft-tissue retractor is used, and, in some cases, it is accompanied by a rib spreader. In men with large areola and women with small breasts a periareolar incision has been utilized (Fig. 1). Ultrasound-guided erector spinae or serratus anterior plane block and surgical site infiltration of relatively long-lasting local anesthetic are always performed before surgical incision.

Two ports are placed in the fourth and sixth intercostal space for 3D thoracoscopy insertion (TIPCAM1TM, Karl Storz, Germany) and carbon dioxide insufflation. Concurrently, an oblique 2-to-3 cm incision is made in the right groin to expose the femoral vessels for cannulation sec. Seldinger technique, and under transesophageal echocardiography guidance. Heparinization is obtained before femoral cannulation. After cannulation, a thorough evaluation of the femoral artery pressure is performed by the perfusionist via fluid administration. If high pressure is detected, the contralateral femoral artery is cannulated, thus bilateral perfusion is obtained. Thoracic fascia bisection is performed during single-lung ventilation using a double-lumen endotracheal tube, that is part of our routine MIMVS anesthetic management together with the percutaneous cannulation of the right internal jugular vein. The pericardium is then opened 2–3 cm above the phrenic nerve and in some cases, two pericardial retraction sutures are passed. A separate aortic root cannula for cardioplegia delivery and venting is placed via the working incision. CPB is then established (vacuum assisted venous drainage is utilized if needed, not exceeding − 40 mmHg), and carbon dioxide is delivered in the mediastinum at 3–4 L/min. The Chitwood clamp is placed through the second interspace at the anterior right axillary line level. Mild hypothermia is targeted, aortic cross-clamp is achieved and antegrade either iso-thermic mixed blood-crystalloid “St. Thomas I” or cold (4 °C) “Custodiol” solution is delivered directly via the aortic root, whereas retrograde cardioplegia is occasionally used. “Custodiol” adoption was mainly related to the use of endovascular balloon occlusion of the Aorta as alternative to transthoracic clamping. Endoartic balloon clamping was the standard of care within the very seminal phase of our minimally invasive MVS experience, whereas it is nowadays adopted almost exclusively in redo cases [7]. The adequacy of surgical results was always confirmed by echographic ascertainment before and after weaning from CPB.

PML prolapse caused by chordal rupture or elongation was treated by scallop resection (quadrangular or triangular) or neochordae implantation depending on surgeon preference. The choice was mainly based on the amount of tissue and the type of degenerative disease: resection was the preferred technique when leaflets width was abundant like in Barlow disease and when preoperative echocardiogram showed increased risk of SAM following repair (sliding technique was sometime associated in these cases); neochords were implanted instead when PML tissue was less abundant like in fibro-elastic deficiency. The choice of the ring size was theoretically conditioned by the type of technique with the use of smaller rings in the resection and larger rings in the neochordae to prevent SAM.

Postoperative management consisted of a multimodal opioid-sparing approach with standardized pain assessments being part of our routine patient evaluation.

## Results

From December 2012 to September 2021, 309 patients underwent endoscopic MVr through right anterolateral mini-thoracotomy. Table 1 describes the baseline characteristics of the population. The mean age was 63 ± 13 years and 99 (32%) were female. The MR etiology was degenerative in 260 (84.1%) patients, secondary in 42 (13.6%), endocarditis in 2 (0.7%) cases, 5 patients had MR due to other causes (i.e., mitral annular disjunction, atrial functional MR). The median EuroSCORE II was 1.12% (0.74–1.99). Four patients had previous cardiac surgery (1.3%). Among patients with degenerative etiology, preoperative echocardiography revealed PML prolapse in 242 patients ( 78.3%), and of the AML in 48 patients (15.5%). Thirty-two patients ( 10.4%) showed a bileaflet prolapse. Thirty-eight patients (15.6%) displayed a concomitant significative TV regurgitation. Preoperative incidence of AF was 27.4%.

**Table 1:** Preoperative characteristics

**Description of the population**	
Cohort	309
Age (years)	63 ± 13
Male gender	210 (68,0)
BMI (Kg/m^2^)	26.2 ± 6.1
NYHA class I II III IV	50 (16.6) 121 (40.2) 130 (43.2) 0 (0.0)
EuroSCORE II (%)	1.12 [0.74–1.99]
**Risk factors**	
Previous cardiac surgery	4 (1.3)
Hypertension	169 (56.1)
Diabetes	27 (9.0)
Dyslipidemia	73 (24.3)
COPD	28 (10.1)
Previous stroke	0 (0.0)
PVD	6 (2.2)
AF	83 (27.4)
Pacemaker-ICD	10 (3.3)
CKD	32 (10.6)
**Echocardiographic features**	
LVEF (%)	56.8 ± 6.1
PAP (mmHg)	34.4 ± 9.4
MV etiology Degenerative Secondary Endocarditis Other	260 (84.1) 42 (13.6) 2 (0.6) 5 (1.6)
MV anulus (mm)	42 ± 4
MV prolapse PML AML Bileaflet	242 (78.3) 48 (15.5) 32 (10.4)
TR 0 1+ 2+ 3+ 4+	18 (7.4) 162 (66.4) 26 (10.7) 31 (12.7) 7 (2.9)

Intraoperative data are shown in Table 2. Mean CPB and cross-clamp time were respectively 126 ± 33 and 93 ± 25 min. Endovascular balloon occlusion of the Aorta was used in 27 cases (9.0%). Cardiac arrest was obtained via administration of blood-crystalloid “St. Thomas I” in 93.3% of cases, whereas “Custodiol” solution was used in the rest of cases. Conversion to full-sternotomy occurred once, due to severe pectus excavatum that rendered the visualization of the MV extremely difficult. Ring annuloplasty was employed in all instances. Concomitant TV repair was performed in 37 cases (12.3%) whereas LAA internal double linear closure, monopolar ablation of AF, and PFO closure were performed respectively in 11 (3.6%), 21 (7.0%), and 6 (2.0%) cases.

**Table 2:** Intraoperative data

Times (min) CPB Cross-clamp	126 ± 33 93 ± 25
**Set up**	
Endoclamp	27 (9.0)
Cardioplegia St. Thomas I Custodiol	280 (93.3) 20 (6.7)
**Surgical technique**	
Ring annuloplasty Isolated	309 (100.0) 49 (15.9)
PML resection With sliding annuloplasty	136 (44.4) 35 (11.4)
Edge-to-edge repair	6 (2.0)
Cleft closure	27 (8.8)
Artificial chords PML AML Both	97 (31.7) 37 (12.1) 9 (2.9)
Other procedures TV repair LAA closure PFO closure Monopolar AF ablation	37 (12.3) 11 (3.6) 6 (2.0) 21 (7.0)
Conversion to full-sternotomy	1 (0.3)

Postoperative results are displayed in Table 3. Intraoperative mortality occurred once (0.3%). Thirty-days mortality was 1.0%. Median mechanical ventilation time and ICU stay respectively were 5 [4–8] hours and 2 [1, 2] days, whereas hospital stay was 6 [6, 7] days. Transfusion rates for whole blood, plasma, and platelets were respectively 22.8% (68), 2.0% [6], and 1.3% [4]. Eight patients (3.0%) needed vasopressors during the postoperative course. Among complications, we experienced bleeding requiring surgical revision in 10 patients (3.3%) (namely, 7 patients had intercostal bleed, 2 patients who underwent transthoracic clamping bled from the aortic needle site, and 1 patient bled from the aortorrhaphy), acute kidney injury requiring dialysis in 9 patients (3.0%). The incidence of POAF was 15.3%, and those of pleural effusion requiring drainage was 4.3%. Other complications occurred very rarely ($\le$1.0% of cases), among these: need for mechanical circulatory support ( ECMO was implanted once for a failure-to-wean from CPB after iatrogenic injury to the Left Circumflex artery), cerebrovascular events, infection, and permanent pacemaker implantation. Echocardiography at discharged demonstrates a freedom from residual MR > 1+ of 99.0%.

**Table 3:** Postoperative results

Mortality Intraoperative 30-days	1 (0.3) 3 (1.0)
Mechanical ventilation time (hours)	5 [4–8]
ICU stay (days)	2 [1–2]
Hospital stay (days)	6 [6–7]
Transfusions Whole blood Plasma Platelets	68 (22.8) 6 (2.0) 4 (1.3)
Vasopressors (> 12 h)	8 (3.0)
Bleeding requiring surgical revision	10 (3.3)
IABP/ECMO	1 (0.3)
Dialysis	9 (3.0)
AMI	1 (0.3)
Stroke	1 (0.3)
Pleural effusion	13 (4.3)
PNX	2 (0.7)
POAF	46 (15.3)
Permanent pacemaker implantation	1 (0.3)
Other arrythmias	5 (1.7)
Infection Respiratory Urinary Thoracic wound	2 (0.7) 0 (0.0) 1 (0.3)
**Echocardiographic features at hospital discharge**	
Residual MR (≤ grade 1)	297 (99.0)
MV mean gradient (mmHg)	3.4 ± 1.2
LVEF (%)	56.8 ± 5.9
Pericardial effusion	2 (0.7)

Follow-up examinations were completed in 278 patients (90.9%), with a mean follow-up period of 28.8 ± 22.0 months (maximum 99 months – 8.3 years) (Table 4). Four patients (1.4%) died during follow-up, none of the deaths was valve-related.

**Table 4:** Follow-up data

Follow-up (months) Follow-up completion	28.8 ± 22.0 90.9
Follow-up mortality	4 (1.4)
MV replacement	2 (0.6)
**Echocardiographic features at follow-up**	
Residual MR 0 1+ 2+ 3+ 4+	38 (13.5) 228 (81.1) 10 (3.6) 4 (1.4) 1 (0.4)
LVEF (%)	57.5 ± 4.6
PAP (mmHg)	29.4 ± 4.9
AMI	0 (0.0)
Stroke	2 (0.7)
Thrombotic events	1 (0.4)
Hemorrhagic events	2 (0.7)
Ex novo AF	9 (3.2)

**Fig. 2:**
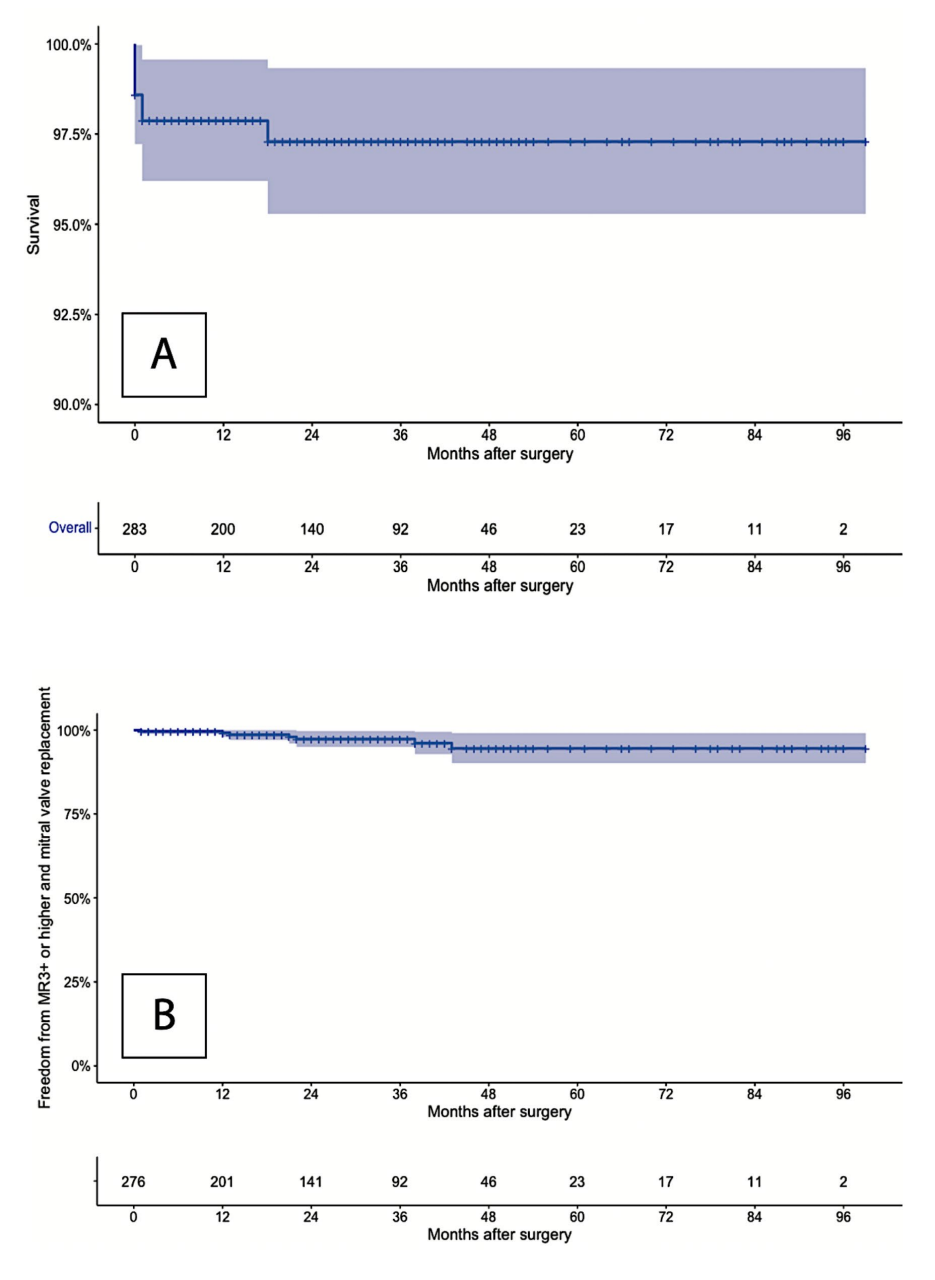
Kaplan-Meier analysis of long-term overall mortality (Panel **A**), and freedom from MV reoperation or MR ≥ 3+ (Panel **B**)

**Fig. 3:**
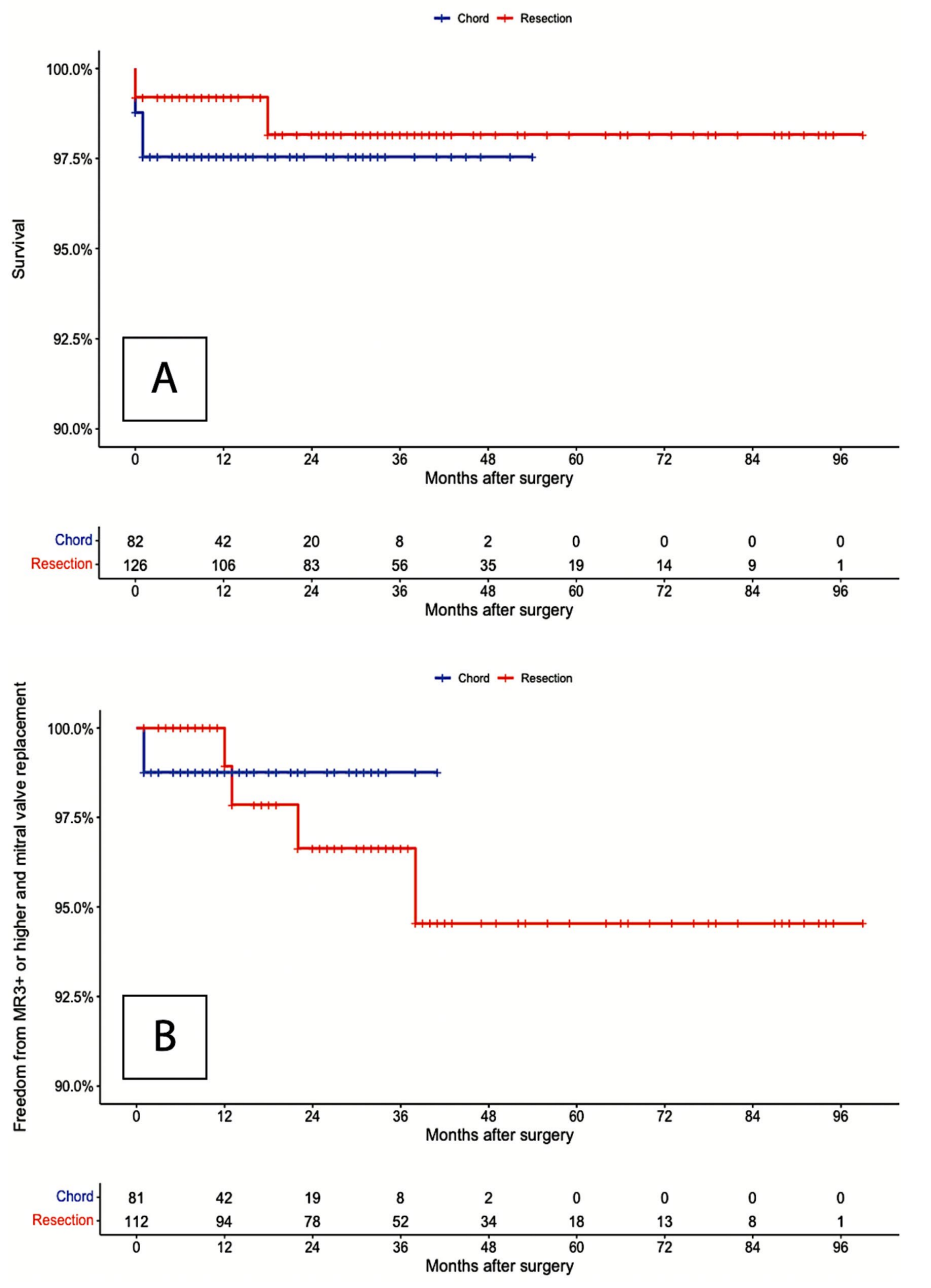
Subgroup Kaplan-Meier analysis for overall survival at follow-up (Panel A), and freedom from MV reoperation or MR ≥ 3 + at follow-up (Panel B)

The overall Kaplan–Meier 5-year survival was 97.3 ± 1.0% (Fig. 2), and the freedom from mitral valve reoperation or residual MR$\ge$3+ was 94.5 ± 2.3% (Fig. 3). Two patients (0.6%) underwent mitral valve replacement during follow-up. Five patients (1.8%) had MR$\ge$3+ at follow-up and were not scheduled for surgical correction after Heart-Team evaluation (i.e., asymptomatic status), a strict follow-up is being rather carried out. The incidence of stroke, thrombotic, and hemorrhagic events was 0.7%, 0.4%, and 0.7% respectively.

Table 2 shows the different MVr techniques. In 242 (78.3) patients PML prolapse was identified at preoperative echocardiography assessment, whereas 210 (68.0%) patients were treated for isolated PML disease after surgical inspection. In 122 (44.4%) patients PML quadrangular o triangular resection was performed, and it was associated in 28 patients with sliding annuloplasty (23.0%). In 88 patients (28.5%), neochords were used instead. These subgroups were analyzed separately (Table 5).

**Table 5:** Subgroup analysis for PML technique

	Resection	Artificial chords	p
Cohort	122	88	
Age (years)	58 ± 12	65 ± 12	**< 0.001**
Male gender	100 (82,0)	62 (70,5)	0.072
BMI (Kg/m^2^)	25.5 ± 4.3	26.3 ± 5.5	0.268
NYHA class I II III IV	30 (24.8) 38 (31.4) 53 (43.8) 0 (0.0)	10 (11.6) 38 (44.2) 38 (44.2) 0 (0.0)	0.058
EuroSCORE II	0.98 [0.68–1.39]	1,11 [0.75–1.76]	0.165
**Risk factors**			
Previous cardiac surgery	0 (0.0)	1 (1.2)	0.171
Hypertension	61 (50.4)	54 (62.8)	0.077
Diabetes	8 (6.6)	10 (11.6)	0.312
Dyslipidemia	25 (20.7)	20 (23.3)	0.656
COPD	10 (10.0)	6 (7.0)	0.638
Previous stroke	0 (0.0)	0 (0.0)	> 0.99
PVD	0 (0.0)	2 (2.4)	0.224
AF	21 (17.4)	18 (20.7)	0.543
Pacemaker-ICD	2 (1.7)	4 (4.7)	> 0.99
CKD	7 (5.8)	10 (11.6)	0.211
**Preoperative echocardiographic features**			
LVEF (%)	58.3 ± 4.6	58.4 ± 3.6	0.900
PAP (mmHg)	33.2 ± 8.5	34.0 ± 9.4	0.551
**Intraoperative data**			
Times (min) CPB Cross-clamp	126 ± 28 94 ± 21	122 ± 38 90 ± 27	0.397 0.211
**Surgical technique**			
Ring size (mm)	34.1 ± 1.7	33.9 ± 1.7	0.366
Edge-to-edge repair	3 (2.5)	0 (0.0)	0.266
Cleft closure	6 (4.9)	11 (12.5)	0.083
Other procedures TV repair LAA closure PFO closure Monopolar AF ablation	5 (4.1) 0 (0.0) 4 (3.3) 6 (5.0)	9 (10.3) 3 (3.4) 1 (1.1) 8 (9.2)	0.138 0.072 0.403 0.268
Conversion to full-sternotomy	1 (0.8)	0 (0.0)	> 0.99
**Early postoperative period**			
Mortality Intraoperative 30-days	0 (0.0) 0 (0.0)	0 (0,0) 0 (0.0)	> 0.99
Mechanical ventilation time (hours)	5 [4–7]	6 [5–9]	0.085
ICU stay (days)	2 [1–2]	2 [1–2]	0.112
Hospital stay (days)	6 [5–6]	6 [6–7]	0.127
Transfusions Whole blood Plasma Platelets	18 (15.0) 2 (1.7) 2 (1.7)	23 (27.1) 2 (2.4) 0 (0.0)	**0.034** > 0.99 0.512
Vasopressors (> 12 h)	2 (2.1)	3 (3.5)	0.668
Bleeding requiring surgical revision	4 (3.3)	2 (2.3)	> 0.99
IABP/ECMO	0 (0.0)	1 (1.2)	0.416
Dialysis	0 (0.0)	6 (7.0)	**0.005**
AMI	0 (0.0)	1 (1.2)	0.416
Stroke	0 (0.0)	0 (0.0)	> 0.99
POAF	11 (9.1)	19 (22.1)	**0.009**
**Echocardiographic features at hospital discharge**			
Residual MR ($\ge$ 3+)	0 (0.0)	1 (1.3)	0.415
MV mean gradient (mmHg)	3.6 ± 1.4	3.4 ± 0.9	0.190
LVEF (%)	58.2 ± 3.9	57,6 ± 4.9	0.384
**Follow-up**			
Follow-up (months) Follow-up completion	35 [18–51] 114 (93,4)	12 [8–24] 82 (95,4)	**< 0.001**
Follow-up mortality	2 (1.6)	2 (2.3)	> 0.99
MV replacement	1 (0.8)	1 (1.1)	> 0.99
**Echocardiographic features at follow-up**			
Residual MR 0 1+ 2+ 3+ 4+	18 (15.5) 91 (78.4) 4 (3.4) 3 (2.6) 0 (0.0)	15 (18.8) 62 (77.5) 2 (2.5) 0 (0.0) 1 (1.3)	0.479
LVEF (%)	58.3 ± 3.8	57.8 ± 3.6	0.240
AMI	0 (0.0)	0 (0.0)	> 0.99
Stroke	1 (0.9)	1 (1.3)	> 0.99
Thrombotic events	1 (0.9)	0 (0.0)	> 0.99
Hemorrhagic events	1 (0.9)	1 (1.3)	> 0.99
De novo AF	2 (1,7)	5 (6.3)	0.122

Preoperative clinical and echocardiographic characteristics were similar as well as early postoperative outcome. No difference was found in terms of implanted annuloplasty ring size, and postoperative mean gradient. Subgroup time-to-event analysis was performed, and opposite curves were compared using the log-rank statistic that showed no statistically significant difference (p = 0.63) (Fig. 3).

## Discussion

Contemporary data arises controversies about the “real-world scenario” of MVS with the enthusiasm for the higher repair rates and lower morbidity associated to less-invasive approaches fading when compared to the actual share of sternal-sparing techniques [2]. Evidence of excellent long-term outcome of MIMVr has been emerging for almost a decade [8–11]. Notwithstanding, it is somehow limited to reference centers worldwide [12–14]. Evidently, detractors’ skepticism regarding the reproducibility of these remarkable long-term results undermined the dissemination of MIMVr. This study provides mid-term outcome of endoscopic MVr performed via a right anterior mini-thoracotomy. Our series demonstrates noteworthy early postoperative results: low 30-days mortality, low incidence of complications and short length of hospital stay, in line with data published in a recent analysis of more than 10,000 patients who underwent MIMVS from the STS ACSD [2]. Furthermore, we report a considerably low stroke rate (0.3%) despite our institutional set up encompasses retrograde perfusion. This is probably determined by the strict arterial line pressure control that is accomplished in all cases. No difference in terms of postoperative stroke rate between conventional sternotomy and less-invasive approaches was also highlighted in retrospective propensity-matched series and well-conducted meta-analyses [15, 16]. Moreover, a recent systematic safety study based on pre-specified 30-days major complications defined by the MVARC reported a 30-days mortality of 1.2% and a stroke rate of 0.3% in a cohort of 745 patients undergoing MIMVS [17]. Hence, our data confirm and even strengthen the available knowledge of early safety and efficacy of MIMVr [8–10]. Our institutional MIMVr set-up also includes percutaneous right internal jugular vein cannulation, thus a double peripheral venous cannulation is chosen over the single femoral cannula approach. We believe this aspect safeguards from undesirable complications like the iatrogenic damage to the right atrium, possibly caused by mispositioning the single venous cannula while exposing the mitral valve. More to the point of safety, we used to exclude obese patients from our endoscopic program during the learning curve. Conversely, we have no absolute exclusion criteria nowadays. Notwithstanding, pectus excavatum is considered a relative criterion requiring precise evaluation.

Interestingly, we report CPB and cross-clamp times of 126 and 93 min, respectively. A finding consistent with other published series [8–15]. The mainstream perception of prolonged surgical times associated with MIMVr fosters the idea of minimally invasiveness as a worthlessly painstaking work. However, a multi-center propensity score matched study showed that this “delay” is limited to 5-to-10 min in dedicated centers [3]. It must also be noted that a reduced thrombo-inflammatory activation is associated with MIMVS as compared to traditional sternotomy, despite significantly longer CPB and cross-clamp times [18].

Long-term favorable outcome and repair durability are the cornerstones of MVr. Assessing the reproducibility of the long-term results obtained with sternotomy is therefore mandatory. We recorded clinical and echocardiographic follow-up data that demonstrate a 5-year survival of 97.3%, and a freedom from replacement or residual MR$\ge$3+ of 94.5%. The Leipzig group has been providing one of the most considerable series of MIMVr with an outstanding long-term follow up that shows a 5-year survival of 87% (decreasing to 74% at 10 years) in a cohort of more than 2,800 patients [13]. Glauber et al. conducted another remarkable follow-up study of 1,137 patients who had MIMVr via right thoracotomy between 2003 and 2013 [19]. They reported a 5-year survival of 93.5% and a 94.8% freedom from reoperation (dropping to 90.1% and 94.8% at 10 years, respectively). McClure et al. published data from a series of 1,000 patients who underwent MIMVS (923 repairs and 77 replacements) [14]. The Boston group reported a 93% 5-year overall survival, and a 96% freedom from reoperation after MIMVr, respectively reduced to 79% and 90% at 15 years. All the more since excellent results have been provided by several cardiac centers worldwide, adopting different less-invasive approaches (e.g., mini-thoracotomies, partial sternotomies, parasternal approaches) and visualization strategies (e.g., direct-vision, video-assistance, robotic), resorting to different reparative techniques (e.g., resection, neochords, edge-to-edge repair, etc.), to treat different pathologies (i.e., mainly degenerative etiology, Morbus Barlow, secondary MR, endocarditis etc.), for different patients (i.e., different age, risk factors, EuroSCORE or STS score, comorbidities, undergoing isolated MIMVr or combined surgery). Paradoxically, the heterogeneity within the available literature represented itself the test bench for MIMVr.

Furthermore, over a decade of MIMVr operations, we have been experiencing a surgical evolution with the most commonly performed technique, namely the Carpentier-type leaflet resection with or without sliding annuloplasty, nowadays being outnumbered by non-resection repairs at our institution. Indeed, we apply the running suture technique described by Tirone David: we pass the suture through the fibrous portion of the papillary muscle then in the free margin of the prolapsing scallop and then again in the papillary muscle with the same needle and suturing the two ends on the free margin.

Since the early reports [20], the chordal replacement has been shown to exceptionally perform by complying with the triad bedrock of MVr described by Alain Carpentier himself almost 40 years ago: preservation of physiologic leaflets motion, large surface of coaptation, and annular stabilization [21, 22]. Peremptory evidence of satisfactory long-term outcome was provided by Tirone David, who pioneered the ePTFE cordal repair technique. David et al. published the results of a 25-year experience encompassing data from 606 consecutive patients treated with ePTFE chordal replacement, mainly used to treat AML prolapse. Mean clinical follow-up was 10.1 years (maximum 23 years), the Kaplan–Meier estimated survival was 98.1%, 85.7%, and 66.8% respectively at 1, 10, and 18 years, whereas the freedom from reoperation at 1, 10, and 18 years was 98.6%, 94.7%, and 90.2%, respectively [23]. The Leipzig group lately provided data of their 15-year experience with more than 2,000 patients undergoing MIMVr. Of these, 1,751 (82.1%) had isolated loop repair. Interestingly, Pfannmueller et al. reported a significantly higher 10-year survival (both all-cause and cardiac) in the “respect” group as compared with those who underwent resection MIMVr, namely 85.6% and 81.0% [12]. Even more recently, Lang et al. published the long-term outcomes of 346 patients who had annuloplasty and chordal replacement for degenerative MR (73% of these with a less-invasive approach), from 2003 to 2010. Mean follow-up was 10.8 years (maximum 15.8 years) and again revealed excellent long-term outcomes, with a 10-year survival of 83.3% together with a low incidence of reoperation of 7.8% [24]. Despite this top-tier evidence, the analysis of the STS ACSD shows that neochords implantation nowadays accounts for just one-third of degenerative MVr [25]. In this landscape, given our progressive shift towards a “respect” approach, we sought to ascertain the possible differences in terms of long-term results between leaflet resection and neochords implantation in a homogeneous cohort of patients (i.e., isolated PML pathology). Finally, our subgroup time-to-event analysis for overall survival and freedom from MR ≥ 3 + showed no differences between the techniques. Interestingly, even if annuloplasty ring of similar sizes were implanted in the two cohorts, no difference in terms of postoperative mean gradient was observed. Indeed, a recent multicenter randomized controlled trial, gainsaid the hypothesis of functional mitral stenosis following leaflet resection versus preservation [26].

However, MVr is not just dichotomous. The answer to the “resect” or “respect” diatribe was perhaps provided by Tirone David, who published his outstanding long-term MVr results from a series of 746 patients with challenging degenerative MR (i.e., bileaflet pathology) [27]. A 20-year cumulative incidence of reoperation with death as a competing risk of 4.2% was reported. Notably, 75% of patients received a combination of chordal replacement and leaflet resection, a percentage that increased to almost 90% in those with bileaflet prolapse. Hence, when dealing with complex pathology (i.e., multisegment or bileaflet disease) it is crucial to get further acquainted with integrating different MVr strategies, in order to guarantee optimal long-term results.

This study has some limitations. The most important one is its retrospective observational design. Moreover, despite our institutional approach to MIMVr has remained relatively unchanged over the last decade (i.e., the time period of the study), some other modifications within our perioperative management (e.g., anaesthetic technique, perfusion materials, general perioperative care) may perhaps represent a potential “treatment era bias”. Furthermore, a progressive shift towards a “respect” MVr over the years was noted and above discussed. Another noteworthy limitation is pertinent to our echocardiographic follow-up that partially relied on external cardiologists, without any standardized protocol for the execution of the examination and the interpretation of the results. Besides, although strongly recommended, not every patient received yearly follow-up echocardiographic examination.

## Conclusions

In our patient cohort, we show that endoscopic MVr via video-assisted right anterior mini-thoracotomy is safe and provides satisfactory postoperative results and excellent mid-term outcomes. Moreover, our subgroup time-to-event analysis showed no differences in terms of survival and freedom from significant MR between patients undergoing resection or neochords implantation for isolated PML disease. Our data is within the range of the existing literature, thus demonstrate the reproducibility of the results from leading institutions worldwide: a paramount step towards the definitive acknowledgement of MIMVr as the gold standard for MR.

## Data Availability

The datasets used and/or analyzed during the current study are available from the corresponding author on reasonable request.

## Abbreviations

AF: Atrial fibrillation
AMI: Acute myocardial infarction
AML: Anterior mitral leaflet
BMI: Body mass index
CKD: Chronic kidney disease
COPD: Chronic obstructive pulmonary disease
CPB: Cardiopulmonary bypass
ECMO: Extracorporeal membrane oxygenation
ePTFE: Expanded polytetrafluoroethylene
IABP: Intra-aortic balloon pump
ICD: Implantable cardioverter-defibrillator
ICU: Intensive care unit
IQR: Interquartile range
LAA: Left atrial appendage
LVEF: Left ventricular ejection fraction
MI: Minimally invasive
MOF: Multiorgan failure
MR: Mitral regurgitation
MVr: Mitral valve repair
MVARC: Mitral Valve Academic Consortium
MVS: Mitral valve surgery
NYHA: New York Heart Association
PAP: Pulmonary arterial pressure
PFO: Patent foramen ovale
PVD: Peripheral vascular disease
PML: Posterior mitral leaflet
PNX: Pneumothorax
POAF: Postoperative atrial fibrillation
SAM: Systolic anterior motion
STS ACDS: Society of Thoracic Surgeons Adult Cardiac Surgery Database
TR: Tricuspid regurgitation
TV: Tricuspid valve
